# Iron metabolism in rheumatic diseases

**DOI:** 10.1016/j.jtauto.2025.100267

**Published:** 2025-01-04

**Authors:** Aliakbar Givian, Amin Azizan, Ahmadreza Jamshidi, Mahdi Mahmoudi, Elham Farhadi

**Affiliations:** a Rheumatology Research Center, Tehran University of Medical Science, Tehran, Iran; b Department of Immunology, School of Medicine, Semnan University of Medical Science, Semnan, Iran; c Research Center for Chronic Inflammatory Diseases, Tehran University of Medical Sciences, Tehran, Iran

**Keywords:** Iron, Rheumatic diseases, Reactive oxygen species (ROSs), Immune system

## Abstract

Iron is a crucial element for living organism in terms of oxygen transport, hematopoiesis, enzymatic activity, mitochondrial respiratory chain function and also immune system function. The human being has evolved a mechanism to regulate body iron. In some rheumatic diseases such as rheumatoid arthritis (RA), systemic lupus erythematous (SLE), systemic sclerosis (SSc), ankylosing spondylitis (AS), and gout, this balanced iron regulation is impaired. Altered iron homeostasis can contribute to disease progression through ROS production, fibrosis, inflammation, abnormal bone homeostasis, NETosis and cell senescence. In this review, we have focused on the iron metabolism in rheumatic disease and its role in disease progression.

## 1 Introduction

Iron is necessary for lots of organisms in terms of cell function and proliferation. Especially, it is required for oxygen transport to tissues through hemoglobin, mitochondrial respiratory chain enzymes, and hematopoiesis. Also, it is crucial for normal immune system function and an imbalance in iron metabolism can disrupt immunity. Hence, the human body has evolved a complex mechanism for iron regulation [1]. Physiologically, iron is absorbed by duodenal enterocytes in the gut. The enterocytes have special transporters on the apical and basolateral membranes that can transfer the iron from the gut to the bloodstream. In the circulation, the iron binds to the transferrin (TF, soluble transporter protein) which carries iron to the cells and tissues. The interaction between TF and TF receptors in the cells can result in the absorption of iron. In addition to gut absorption, the recycling of iron that is stored in red blood cells (RBCs) can be a source of iron. Iron excretion can occur through intestinal epithelial cell shedding, menstruation, or skin desquamation [[2], [3], [4]].

Because iron is vital in microbial cell division and proliferation, the body (for fighting infections) limits iron availability for pathogens through the induction of inflammation. Inflammation-mediated iron restriction can also occur in the case of inflammatory chronic diseases such as rheumatic diseases that can affect immunity [1,2]. This type of iron deficiency named "functional iron deficiency" is caused by proinflammatory mediators and hepcidin which is a key regulator of iron metabolism. Hepcidin reduces circulating iron by suppressing ferroportin (FPN, an iron exporter) function and arrests iron in stores [3].

Autoimmune rheumatic diseases are characterized by autoantibody formation and inflammation. Studies have shown that rheumatoid arthritis (RA), systemic lupus erythematosus (SLE), systemic sclerosis (SSc), ankylosing spondylitis (AS), and gout are associated with altered iron homeostasis. In some of them, reactive oxygen species (ROS) resulting from altered iron homeostasis can play an important role in disease progression [2].

In this review, we have a discussion about iron homeostasis in rheumatic diseases and the role of altered iron metabolism in the pathogenesis of rheumatic diseases (Fig:2, Fig:3).

**Fig:1:**
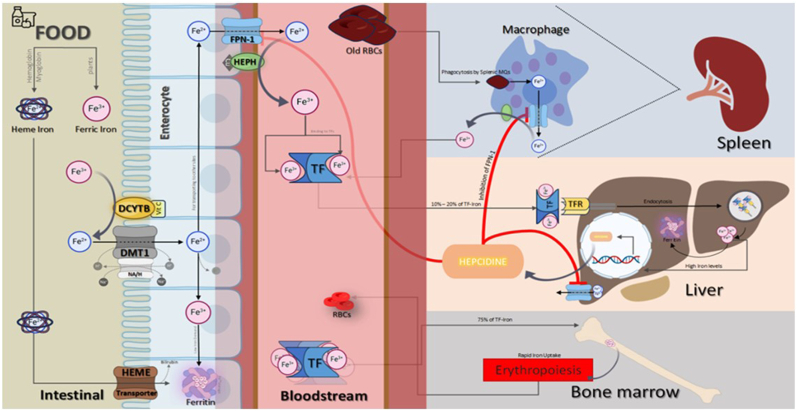
**Mechanism of Iron Absorption and Regulation in the Human Body.** Iron is absorbed in the duodenum by enterocytes, primarily in the Fe²⁺ form, through the DMT-1 located on the apical membrane. Ferric iron (Fe³⁺) is first reduced to Fe²⁺ by DcytB before absorption. Once inside the enterocyte, iron can either be exported into circulation by FPN-1 on the basolateral membrane or stored as FT. In the bloodstream, iron is oxidized by hephaestin or ceruloplasmin and binds to TF, facilitating its transport to target cells. Macrophages in the spleen, play a critical role in recycling iron by phagocytosing aged or damaged RBCs and releasing the iron into the bloodstream. This recycled iron is then transported via transferrin to target organs, such as the liver and bone marrow. In the liver, iron is stored or used for metabolic processes, while in the bone marrow, iron is essential for erythropoiesis, where it is utilized in the synthesis of hemoglobin during red blood cell production. DCYTB; Duodenal Cytochrome B, DMT1; Divalent Metal Transporter 1, FPN-1; Ferroportin 1, HePH; Hephaestin, TF; Transferrin, RBC; Red Blood Cells, TFR; Transferrin Receptor, CU; Copper, FT; Ferritin

**Fig:2:**
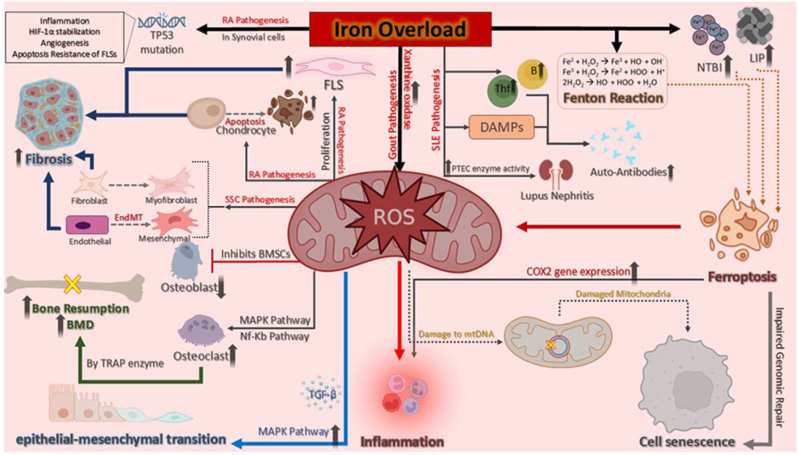
**The role of iron overload (IO) and reactive oxygen species (ROS) in the pathological mechanisms associated with rheumatic diseases.** IO leads to LIP in cells and NTBI in circulation, which promotes oxidative stress through the Fenton reaction, generating OH˙. These ROS can induce various forms of cell injury, including ferroptosis, and contribute to fibrosis by enhancing TGF-β-induced EMT. Excess iron also promotes osteoporosis by stimulating osteoclast differentiation via the MAPK and NF-κB pathways while inhibiting osteogenic differentiation of BMSCs. Additionally, IO induces apoptosis in chondrocytes, leading to cartilage damage. In RA, IO-mediated ROS can cause p53 mutations, resulting in defective p53 function, which promotes inflammation, angiogenesis, and apoptosis resistance. IO in SLE enhances Tfh cell differentiation, GC B cell expansion, and autoantibody production, leading to lupus nephritis. In SSc, iron contributes to fibrosis by promoting EndMT and fibroblast-to-myofibroblast transition. In gout, IO activates XO, increasing ROS production and contributing to inflammation. IO; Iron Overload, LIP; Labile Iron Pool, NTBI; Non-Transferrin Bound Iron, OH˙; Hydroxyl Radical, TGF-β; Transforming Growth Factor-Beta, EMT; Epithelial-to-Mesenchymal Transition, MAPK; Mitogen-Activated Protein Kinase, NF-κB; Nuclear Factor Kappa B, BMSCs; Bone Mesenchymal Stem Cells, RA; Rheumatoid Arthritis, FLSs; Fibroblast-Like Synoviocytes, SLE; Systemic Lupus Erythematosus, Tfh; T Follicular Helper Cells, GC; Germinal Center, LN; Lupus Nephritis, DAMP; Damage-Associated Molecular Pattern, SSc; Systemic Sclerosis, EndMT; Endothelial-Mesenchymal Transition, XO; Xanthine Oxidase

**Fig:3:**
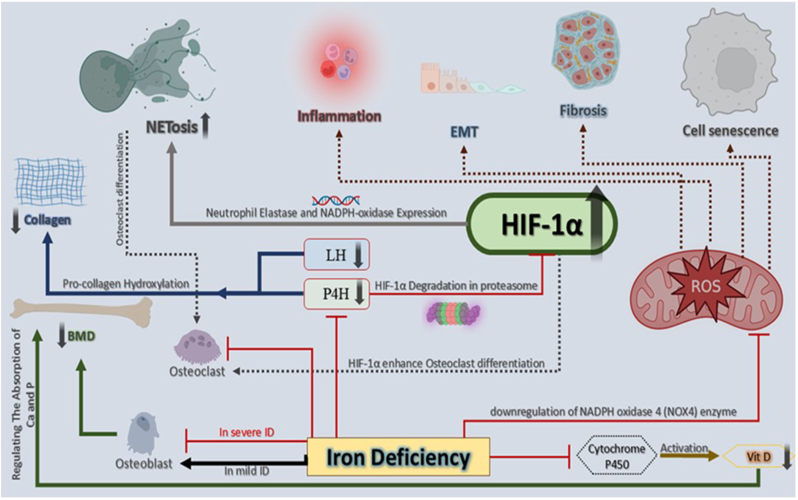
**The role of iron deficiency (ID) in the pathological mechanisms associated with rheumatic diseases.** ID has been associated with reduced BMD and impaired bone matrix formation due to its role as a cofactor for prolyl-4-hydroxylase (P4H) and lysyl-hydroxylase (LH), enzymes involved in collagen synthesis. While mild ID may promote osteoblast differentiation, severe ID inhibits osteoblast function and bone formation. ID also reduces ROS production by inhibiting the Fenton reaction and downregulating NOX4, leading to decreased osteoclast differentiation. However, iron chelation prevents prolyl-4-hydroxylase activity, which stabilizes HIF-1α by inhibiting its proteasomal degradation, promoting osteoclast activity and bone resorption. In the context of NETs formation, ID increases NETosis via HIF-1α stabilization, which upregulates neutrophil elastase and NADPH oxidase expression, while HRE in the promoter region of PAD4 further enhance NET formation. Heme can also stimulate NET generation through ROS and heme-iron interactions. Thus, iron homeostasis is essential for both bone health and neutrophil function, particularly NETosis. ID; Iron Deficiency, BMD; Bone Mineral Density, ROS; Reactive Oxygen Species, NOX4; NADPH Oxidase 4, HIF-1α; Hypoxia-Inducible Factor 1-Alpha, NETs; Neutrophil Extracellular Traps, PAD4; Peptidyl-Arginine-Deiminase 4, HRE; Hypoxia-Responsive Elements.

## 2 Iron metabolism in a physiologically healthy state

In the human body, iron absorption occurs in the duodenum by the intestinal epithelial cells named "enterocytes". This metal may be in ferrous (Fe^2+^) or ferric (Fe^3+^) form. Fe^2+^ is absorbed by the divalent metal transporter-1 (DMT-1) which is a special transporter on the apical-luminal membrane of the enterocytes and some other cell types. Fe^3+^ cannot directly enter through this transporter therefore firstly must be reduced to Fe^2+^ catalyzed by the transmembrane enzyme, duodenal cytochrome B ferrireductase (DcytB), located on the apical side of the enterocytes. Depending on body requirements, enterocyte iron can be exported into the circulation by ferroportin-1 (FPN-1) which is located on the basolateral membrane of the enterocytes (and some other cell types), or can be stored in the ferritin (FT) form. The storage protein, FT, keeps oxidized iron in a soluble and relatively non-toxic form in the cells. The exported iron, in the circulation, is oxidized by the ferroxidase enzyme "ceruloplasmin" or its enterocyte equivalent "hephaestin" and then binds to the TF that can bind two Fe^3+^ molecules. TF moves iron in circulation. TF receptors on the hepatocytes (HCs), macrophages, erythroid precursor cells, and other cell types regulate TF-iron uptake through endocytosis of the TF-TF receptor complex. In these cells, if iron is not needed for metabolic requirements, it is stored as FT [[2], [3], [4]]. Inside cells, the chelatable iron that is not bound to proteins forms a labile iron pool (LIP) that can participate in ROS production and lead to cellular damage [5].

The hepcidin-FPN-1 axis is responsible for systemic iron homeostasis. When there is excessive iron in the circulation, HCs produce hepcidin which in turn suppresses FPN-1 function and reduces iron release from the enterocytes, macrophages, and HCs into the circulation [6,7] (Fig. 1).

## 3 Role of iron in the immune system

Iron has a crucial role in cell growth and differentiation and is an important component of the numerous enzymes that are necessary for the enzymatic activity of the immune cells (for example peroxides, nitric oxide synthase (NOS), and those involved in cytokine production) [2]. In innate immunity, iron adjusts the myeloid cell function because it tunes the activity of transcriptional factors and enzymes and thus the production of antimicrobial substances like nitric oxide (NO) and hydroxyl radicals (OH°). In adaptive immunity, iron is a vital growth factor for lymphocyte clonal expansion [[8], [9], [10]]. Also, it has been shown that the expression of the TF receptor is significantly increased on the cell surface of activated CD4^+^ and CD8^+^ T cells which indicates the vital role of iron in the T cell activation [11].

### 3.1 Iron deficiency

It has been revealed that ID affects memory and effector T cell responses to the vaccine which is impaired in the mice with ID [12]. While all of the lymphocytes appear to need the transferrin-iron uptake, it seems that the Th1 subset is more sensitive to altered iron homeostasis [13]. Transferrin-iron uptake is essential for Th1 cell proliferation. Iron chelation inhibits Th1 cell proliferation [14,15] and iron supplementation disrupts T-bet (Th1 cell master transcription factor) expression [16], so it seems that an optimal concentration of iron is necessary for Th1 proliferation. Th2 cells are less sensitive to the altered iron homeostasis compared with Th1 thus in the ID conditions, Th polarization switches towards Th2, Furthermore, ID can impair the B cell functions such as the IgM production [1].

Iron chelation suppresses the expression of the forkhead box protein 3 (Foxp3, Treg cell master transcription factor), CD25 level, and phosphorylation of the signal transducer and activator of transcription 5 (STAT5) [[17], [18], [19], [20], [21]]. Also, it has been reported that iron-metabolizing heme oxygenase 1 (HO-1) is an important enzyme for Treg function [18,19,21].

Iron chelation, in the macrophages, stimulates the hypoxia-inducible factor (HIF)-1α and nuclear factor (NF)-IL6 thus inducing NO production by the NOS. In contrast, iron chelation inhibits the OH° production in the macrophages. Besides, ID induces metabolic reprogramming in the macrophage energy metabolism in a way that reduces mitochondrial oxidative phosphorylation and increases glycolysis [22].

Overall, iron deficiency (ID) decreases T-lymphocyte proliferation, the number of T helper cells (Th) and cytotoxic T cells (CTL) in blood, bactericidal activities of neutrophils and macrophages, reduces TNF and IL-2 production, decreases IFN-γ, IL-12, IL-2, IL-6, and IL-10 levels in human serum, and decreases humoral, cell-mediated and non-specific immunity responsiveness to functional tests of immune function [2].

### 3.2 Iron overload

Iron overload (IO) due to hereditary hemochromatosis (HH) results in reduced CD8^+^ T cell numbers and a divergent T cell receptor (TCR) repertoire. IO causes continuous activation and subsequent exhaustion of CD8^+^ T cells which is associated with a reduced cytotoxicity [[23], [24], [25], [26]].

Several studies have shown that IO prevents the function of myeloid cells. Iron suppresses the transcription of MHC-II and ICAM-1, which can lead to impaired interaction between Th1 cells and macrophages and reduced antigen presentation by macrophages [27]. Although a certain amount of iron is necessary for sufficient translation of TNF and other cytokines [28] and antimicrobial oxidative stress responses [29,30], high intracellular iron inhibits the transcription of TNF [31], NOS2 induction, and tryptophan degradation in human monocytes [32,33].

IO can increase the number and activity of the suppressor T cells, alter immunoglobulin (Ig) secretion [34], and reduce the production of proinflammatory cytokines such as IL-2, IFN-γ, and TNF which can lead to decreased T cell proliferation. In addition, IO reduces granzyme-B and perforin production and increases the apoptosis of CTLs possibly mediated by iron-induced ROS generation [35].

IO inhibits the production of NO and TNF in macrophages and prevents the immunoglobulin class from switching towards IgG in B cells [1]. IO shifts the immune balance toward the Th2 [36] which could be reversible by using the iron chelator [37]. It seems that both ID and IO inhibit Th1 and an optimal concentration of iron is needed for balanced Th1.

## 4 Impact of the immune system mediators on iron metabolism

Pro-inflammatory cytokines including TNF, IL-1, IL-6, and IL-22 are known as modifiers of iron metabolism [[38], [39], [40], [41], [42]]. IL-6 increases hepcidin and decreases TF production by HCs [43,44] which results in reduced levels of iron in plasma (hypoferremia) and retention of iron in the macrophages and enterocytes [1]. TNF, IFN-γ, and IL-10 increase DMT-1 and TF receptor-1 expression on the macrophages thus increasing iron uptake [39]. In addition, the anti-inflammatory cytokines IL-4 and IL-13 increase the TF receptor-1 mRNA expression and FT translation thus contributing to iron sequestration in myeloid cells [45]. Also, IL-1β and IL-6 turn on FT expression and translation that contribute to iron sequestration in macrophages [41,46].

## 5 Iron and cellular processes

### 5.1 Oxidative stress

Oxidative stress refers to the condition in which there is a disturbance in the cellular prooxidant-antioxidant balance in favor of ROS production [47,48]. In aerobic organisms, consumed O_2_ is physiologically reduced to H_2_O by the mitochondrial respiratory chain complex IV [49]. However, a small portion of O_2_ can be "partially" reduced during mitochondrial respiratory chain reactions or biochemical activities such as phagocytosis, immune activation, and xenobiotics metabolism [50]. This leads to production of the harmful intermediates such as superoxide anion (O_2_^−^), hydrogen peroxide (H_2_O_2_), and hydroxyl radical (OH˙) generally called reactive oxygen species (ROS). O_2_^−^ and H_2_O_2_ are moderate and OH˙ is an extremely reactive species [51]. It is worth noting that, under physiological conditions, O_2_^−^ and H_2_O_2_ are permanently produced and removed in the cells [52].

The existence of iron in two forms (Fe^2+^ and Fe^3+^), its converting capacity between two forms, and its ability to receive or provide electrons result in the participation of iron in important biochemical reactions. On the other hand, these abilities have made iron a catalyst for the generation of ROS in aerobic organisms. Most iron in the circulation is bound to the TF in inactive and protected form. Also, in the cells iron is stored as FT in inactive and protected form. Nevertheless, there is always a small proportion of iron that is free in the circulation (NTBI, non-TF-bound iron) and in the cells (LIP) and can participate in redox reactions.

Although O_2_^−^ and H_2_O_2_ are not reactive enough to oxidize cellular macromolecules such as nucleic acids, proteins, membrane lipids, and carbohydrates, the presence of free and active iron can promote the “Fenton reaction” and contribute to excessive generation of extremely reactive OH˙ radical (reaction 1) [53]. In pathologic states such as IO due to excess consumption or impaired excretion or some chronic inflammatory conditions, there is increased LIP in the cells or increased circulating NTBI [54,55]. This excess iron can promote the Fenton reaction and produce excessive OH˙ radicals. OH˙ is a strong reactive molecule that can oxidize any chemical group (in the macromolecules) [56]. This oxidative stress can result in cell injuries including ferroptosis (an iron-dependent form of cell death) [51]. Studies have shown that ferroptotic cell death is associated with excessive labile iron levels and production of excessive free radicals (which is extremely reactive) and membrane phospholipids peroxidation (specifically arachidonic and adrenic acids in the phosphatidyl ethanolamine (PE)) [57,58].

Reaction1) Fe^2+^ + H_2_O_2_ Fe^3+^ + OH˙ + OH ^־^

### 5.2 Fibrosis

Fibrosis refers to the overproduction and excessive accumulation of extracellular matrix (ECM) components such as collagen and is related to the activated fibroblast function. Indeed, it is a dysregulated tissue repair reaction to tissue injuries including chronic inflammatory disorders [59]. Although there is no clear known mechanism underlying fibrosis, epithelial-mesenchymal transition (EMT) has been proposed as a possible mechanism of fibrosis [60]. EMT is a process in which epithelial cells undergo transition to the mesenchymal cells such as fibroblasts and myofibroblasts (activated fibroblasts) involved in fibrosis. Transforming growth factor-β (TGF-β) is a critical factor for EMT and fibroblast activation [61].

On the other hand, recent data have revealed that iron metabolism may regulate TGF-β-induced EMT by ROS production [62]. A study has reported that ROS may play a part in TGF-β1-induced EMT through activation of mitogen-activated protein kinase (MAPK) and antioxidants can inhibit EMT in renal tubular epithelial cells [63]. Also, a study has revealed that iron chelation and modification of mitochondrial dysfunction reduces TGF-β-induced EMT in an alveolar epithelial cell line and transbronchial iron chelation mitigated bleomycin-induced pulmonary fibrosis in a mouse model [62]. Another study has shown the inhibitory effect of iron chelation on lung fibrosis *in vivo* [64]. Therefore, it is claimed that IO-induced ROS may contribute to fibrosis through EMT induction.

### 5.3 Inflammation

As mentioned above, macrophages are important stores of iron but turnover of this metal is different in M1 (pro-inflammatory subtype) and M2 (anti-inflammatory subtype) macrophages. M1 macrophages express lower FPN and hemoxygenase-1 (HO-1) and subsequently have more amount of intracellular iron and FT. M1 cells are more susceptible to iron accumulation compared to M2 cells. M2 macrophages possess the ability to metabolize and export iron and thus have lower intracellular iron concentrations [65]. The level of intracellular iron in macrophages can be an important element in macrophage polarization [66]. A low intracellular iron level inhibits pro-inflammatory cytokine production [28,67] while a high intracellular iron level promotes the pro-inflammatory setting [68,69].

On the other hand, some studies have suggested that macrophage iron depletion may have pro-inflammatory effects [70]. It has been revealed that Iron depletion in the macrophages can stabilize HIF-1α and subsequently promote IL-1β expression and synthesis [71]. IL-1β contributes to systemic inflammation [72].

In addition, excessive ROS production (in the case of excess iron) can result in inflammasome activation and consequent inflammation [73].

### 5.4 NETosis

NETosis is the releasing of neutrophil extracellular traps (NET) which are web-like structures from neutrophils. NETs contain decondensed chromatin DNA and contents of neutrophil granules such as myeloperoxidase (MPO) and neutrophil elastase (NE) [[74], [75], [76]]. Neutrophils release the NETs to eliminate invading pathogens. However, despite their beneficial role in infectious diseases, NETs can promote some medical conditions such as autoimmune diseases, thrombosis, and cancer metastasis [77]. The key mechanisms that contribute to NETosis consist of the NADPH-dependent formation of ROS [78], neutrophil elastase-mediated histone degradation [76], and the peptidyl-arginine-deiminase 4 (PAD4)-mediated histone hypercitrullination [79].

Some reports have revealed that iron restriction can increase NETosis in the neutrophils [80,81]. The possible underlying mechanism is that under ID status HIF-1α will not be degraded because of the inhibition of prolyl hydroxylase function, which requires iron and leads to HIF-1α stabilization. HIF-1α increases neutrophil elastase and NADPH-oxidase expression on the transcriptional level [82]. Besides, hypoxia-responsive elements (HRE, HIF-1α specific binding site) are found in the promoter region of the PAD4 enzyme [83]. Therefore, HIF-1α may lead to the activation of enzymes involved in triggering NET formation by iron chelation or restriction [81]. Furthermore, it has been shown that heme stimulates neutrophils to generate NETs *in vitro* which is associated with ROS and heme-iron [84]. Thus, it seems that an optimal iron homeostasis is required for neutrophil function and NETosis.

### 5.5 Senescence

Although some reports suggest that iron accumulation in senescent cells is a consequence of senescence [85], other studies mention that excess iron can be a leading cause of senescence. Cellular senescence refers to irreversible cell cycle arrest induced by signals such as telomere shortening and DNA damage [86]. Senescent cells are characterized by high macromolecular (including DNA) damage, altered metabolism, and specific secretory phenotype [[87], [88], [89], [90]]. Although senescence has beneficial roles for organisms e.g., prevention of unwanted cell proliferation [86,89], accumulation of senescent cells in tissues may lead to tissue dysfunction, inflammation, and tumorigenesis [[89], [90], [91]]. As mentioned above, excess iron can lead to the generation of ROS. Excess iron in the cells can lead to DNA damage via the Fenton reaction and impaired genomic repair system, resulting in cellular senescence [92]. Also, IO can contribute to mitochondrial dysfunction. Studies have shown that iron accumulation in mitochondria can result from frataxin deficiency, which plays an important role in iron homeostasis in mitochondria and can result in oxidative stress and consequently apoptosis or cellular senescence through mitochondrial dysfunction [[93], [94], [95], [96]]. ROS can harm mitochondrial DNA (mtDNA) and lead to mutation in mtDNA and mitochondrial dysfunction [97]. In another study, it has been revealed that intracellular iron chelation with DFO suppresses ROS-induced senescence in human-endometrium-derived mesenchymal stem cells (hEMSCs) [98]. In addition, it has been reported that the presence of excess iron and the production of ROSs cause the accumulation of lipofuscin, which is a hallmark of senescence [[99], [100], [101]].

### 5.6 Ferroptosis

Ferroptosis is an iron-dependent form of regulated cell death, distinct from apoptosis and necroptosis, and is characterized by the accumulation of lipid peroxides to toxic levels. This process is driven by iron-mediated oxidative damage to membrane lipids, primarily polyunsaturated fatty acids, through the production of ROS via the Fenton reaction [[102], [103]]. Dysregulated iron metabolism, such as increased iron uptake, ferritin degradation, or impaired iron export, amplifies cellular vulnerability to ferroptosis [104]. Additionally, the depletion of antioxidant defenses, such as glutathione (GSH) and glutathione peroxidase 4 (GPX4), exacerbates lipid peroxidation and accelerates ferroptotic cell death [105].

Iron overload has been identified as a primary driver of ferroptosis, with dysregulation of iron absorption, storage, and utilization contributing to increased cellular susceptibility [106]. Key regulatory proteins such as hepcidin and ferroportin control systemic iron homeostasis. Hepcidin binds to FPN, reducing iron absorption by promoting its degradation, while hypoxia-inducible factors (HIFs) suppress hepcidin expression, enhancing iron release [107,108]. Excess non-transferrin-bound iron (NTBI) accumulates in unstable pools, amplifying oxidative stress and triggering ferroptosis [109]. Therapeutic interventions, including iron chelators like deferoxamine (DFO) and FPN inhibitors, are under investigation to mitigate iron-driven ferroptosis [110,111].

Moreover, lipid peroxidation is a hallmark of ferroptosis, where ROS oxidize polyunsaturated fatty acids (PUFAs) within phospholipids, disrupting membrane integrity [112]. Enzymes such as acyl-CoA synthetase long-chain family member 4 (ACSL4) and lysophosphatidylcholine acyltransferase 3 (LPCAT3) are pivotal in incorporating PUFAs into membrane lipids, making them more susceptible to peroxidation [113]. Conversely, monounsaturated fatty acids (MUFAs), regulated by ACSL3, can inhibit ferroptosis by preventing lipid ROS accumulation [114]. Additionally, lipoxygenases (LOXs), a family of iron-containing enzymes, directly oxidize PUFAs, further driving lipid peroxidation and ferroptosis [115].

Mitochondrial dysfunction also contributes to ferroptosis, with excessive ROS production from the electron transport chain damaging mitochondrial membranes and inducing lipid peroxidation [116]. Additionally, hypoxia-induced lipid remodeling and immune signaling molecules like interleukin-4 (IL-4) and IL-13 further modulate ferroptosis susceptibility by altering lipid metabolism and GPX4 expression [117]. In addition, there is a complex relationship between ferroptosis and arachidonic acid (AA) metabolism and eicosanoids synthesis which can connect inflammation to iron. The evidence shows that ferroptosis induces the expression of the prostaglandin-endoperoxide synthase 2 (PTGS2) gene which encodes cyclooxygenase 2 (COX-2) enzyme thereby accelerating AA metabolism and increases pro-inflammatory signaling molecules secretion [118]. The activity of COX and LOX (lipoxygenase) enzymes is under control of intracellular lipid peroxides level [119,120]. As mentioned above, ferroptosis is accompanied by oxidized lipids release and can induce the activity of COX and LOX enzymes. Besides, ferroptosis (unlike apoptosis) may happen to a proinflammatory state by the release of damage-associated molecular patterns (DAMPs) such as HMGB-1 (High mobility group box 1 protein) [121]. Also, increased intracellular iron levels can contribute to apoptosis and ferroptosis in BMSCs [122].

Ferroptosis, a form of regulated cell death, has emerged as a significant factor in Rheumatoid arthritis (RA), Systemic lupus erythematosus (SLE) and Myasthenia gravis (MG) pathogenesis due to its involvement in inflammatory responses, oxidative stress, and immune dysfunction [112].

Disrupted iron metabolism contributes to RA development by inducing oxidative damage, impairing immune cell function, and promoting chronic inflammation. Central features of ferroptosis, such as glutathione peroxidase 4 (GPX4) inactivation, glutathione (GSH) depletion, lipid peroxidation, and iron accumulation, are increasingly recognized in RA [123].

Recent studies have identified ferroptosis as a significant mechanism underlying neutrophil death in SLE. Morphological characteristics of ferroptosis in neutrophils include vacuole formation, loss of mitochondrial cristae, and increased mitochondrial membrane density [124]. Furthermore, GPX4 exhibits diminished expression in neutrophils of SLE patients, though its levels remain unchanged in other immune cells. Interestingly, myeloid-specific GPX4-haploinsufficient mice develop spontaneous lupus-like symptoms, whereas complete GPX4 ablation in neutrophils results in severe neutropenia without inducing lupus-like disease [124]. Notably, treatment with liproxstatin-1, a ferroptosis inhibitor, significantly attenuates disease progression in SLE models [125]. Ferroptosis also impacts other immune cells in SLE. For instance, studies indicate that ferroptosis inhibitors can regulate the TH1/TH2 cytokine balance, thereby modulating disease progression in SLE mouse models [126]. Peripheral blood monocytes in SLE patients also exhibit ferroptotic features, such as reduced mitochondrial volume, increased membrane density, and the absence of mitochondrial cristae [127].

Disrupted iron homeostasis in MG correlates with increased ROS production, reduced protective autophagy, and skeletal muscle death [128]. Indicators of iron metabolism, such as serum iron levels, inversely correlate with IL-6 and anti-AchR antibody levels, highlighting their potential as biomarkers for disease severity and therapeutic efficacy [129].

### 5.7 Endoplasmic reticulum stress

The endoplasmic reticulum (ER) plays a vital role in protein folding, lipid metabolism, and calcium regulation, and its function is disrupted during conditions like oxidative stress, hypoxia, and inflammation [130,131]. This disruption can trigger the unfolded protein response (UPR), which modulates iron metabolism through alterations in the expression of key iron-related genes, including hepcidin, ferroportin, and ferritin H [132]. Studies demonstrate that ER stress can stimulate hepcidin production via pathways involving C/EBPα and CREBH activation, highlighting a bidirectional link between UPR and iron homeostasis [133]. Additionally, calreticulin (CRT), a key ER chaperone, protects against iron-induced oxidative stress and contributes to MHC-I assembly, potentially explaining its regulatory role in iron overload conditions such as hemochromatosis [134].

Moreover, Recent studies highlight intricate connections between ferroptosis and ER stress, with significant implications for various diseases, including autoimmune conditions [135].

In RA, the cationic channel TRPM7 has been implicated in Ca2+-mediated ERS and ferroptosis [136]. Inhibition of TRPM7 reduces intracellular Ca2+ overload, attenuates PKCα/NOX4-dependent ROS production, and mitigates cartilage destruction and synoviocyte apoptosis in experimental arthritis models [137,138]. These findings suggest a complex and context-dependent interaction between iron metabolism and ERS, warranting further investigation, particularly in autoimmune diseases like RA.

## 6 Influence of iron on bone homeostasis

The bone is a dynamic tissue that continuously is formed and reabsorbed. Bone remodeling and homeostasis depend on two major cell types: osteoclasts (bone-resorbing cells) and osteoblasts (bone-synthesizing cells). Both IO and ID are associated with bone loss [139].

### 6.1 Iron overload

IO is regarded as a serious risk factor for osteoporosis. The studies have revealed that there is an association between IO and osteoporosis occurrence in some diseases such as thalassemia [140] and hemochromatosis [141]. IO can result in osteoporosis by promoting bone resorption and inhibition of bone formation. ROSs play a role in the osteoclast differentiation by the MAPK and NF-κB signaling pathway activation [142]. It has been shown that bone loss in an IO model was linked to elevated ROS levels and inhibition of ROS production could partially reduce the bone loss in this model which indicates the important role of ROS in bone loss [143]. Also, osteoclasts express TRAP (tartrate-resistant acid phosphatase) enzyme which dephosphorylates some bone matrix proteins and contributes to bone matrix degradation [144]. Some studies reported that inhibition of TRAP in osteoclasts attenuates bone resorption and increases bone mineral density (BMD) [145,146]. TRAP is an iron-containing enzyme whose activity depends on the iron [[147], [148], [149]]. On the other hand, excess iron inhibits bone mesenchymal stem cells (BMSCs, osteoblast precursors) osteogenic differentiation. This inhibitory effect has been proven by the downregulation of runt-related transcription factor 2 (RUNX2, master transcription factor for osteogenesis and osteoblastic marker) and its downstream molecules alkaline phosphatase and osteocalcin [150]. This inhibitory effect seems to depend on the up-regulation of FT [150] because osteoblasts respond to the IO by downregulation of TF receptor and up-regulation of FT (light and heavy chain) [151] and this elevated expression of FT, especially ferroxidase activity of heavy chain subunit, seems to have an important role in iron-mediated inhibition of osteoblast activity [152,153]. Iron chelation by deferoxamine (DFO) increases osteoblast differentiation by induction of Wnt5a [154,155]. DFO inhibits excess iron-induced caspase-3 expression and apoptosis in BMSCs by the suppression of ROS production [156].

### 6.2 Iron deficiency

The impact of ID in bone homeostasis is less understood but some studies have shown that ID leads to a significant reduction of BMD [[157], [158], [159]]. Although some reports show that mild ID stimulates osteoblast differentiation. Severe ID has an inhibitory effect on osteoblastic differentiation [160]. Iron is a crucial cofactor for prolyl-4-hydroxylase and lysyl-hydroxylase enzymes which catalyze hydroxylation of pro-collagen and lead to collagen synthesis (an important component of bone matrix) [161,162]. In addition, iron is a vital element for the cytochrome P450 enzyme family which plays a role in vitamin D activation [163]. Active vitamin D plays a part in bone homeostasis by regulating the absorption of calcium and phosphate [164]. During osteoclastogenesis, osteoclast precursors need a high amount of iron to supply appropriate mitochondrial functions. In addition, to bone resorption, osteoclasts require high energy and consequent abundant mitochondria and iron supply [165,166]. Therefore, iron chelation can inhibit osteoclastogenesis and bone resorption [167]. Free iron chelation decreases ROS level by the inhibition of the Fenton reaction [168] and therefore leads to the inhibition of osteoclast differentiation. Also, iron chelation can decrease ROS by the downregulation of NADPH oxidase 4 (NOX4) enzyme (a main source of ROS production) [156,169] and consequently inhibits osteoclast differentiation. Furthermore, prolyl-4-hydroxylase (an iron-dependent enzyme) activity results in HIF-1α degradation in proteasome, and therefore iron chelation prevents HIF-1α degradation and leads to persistent HIF activation [170]. The reports have shown that HIF activation increases bone resorption by osteoclasts [[171], [172], [173]]. This mechanism may play a part in bone loss associated with chronic ID [174].

Overall, most of the data show that iron chelation leads to inhibition of osteoclastogenesis and induction of osteoblast differentiation.

Most iron-related therapies in RA primarily address anemia associated with the disease [175,176]. However, emerging studies indicate that iron and iron-modulated pathways may offer innovative therapeutic strategies for RA. Ferroptosis, an iron-dependent form of lipid peroxidative cell death, has shown significant potential for targeting infiltrated inflammatory cells and proliferated FLS in RA. Two similar studies demonstrated the use of macrophages as vectors to deliver Fe₃O₄ nanoparticles and sulfasalazine (SSZ) for combined ferroptosis and photothermal therapy. These macrophages, guided by inflammatory signals, migrated to inflamed joints, where near-infrared light irradiation activated the Fe₃O₄ nanoparticles. The resulting heat damaged the synovium, and the released iron synergized with SSZ to induce ferroptosis [177,178].

These insights highlight the potential of integrating ferroptosis-targeted approaches into RA management, paving the way for innovative and effective treatments.

## 7 Role of iron in rheumatoid arthritis (RA) pathogenesis

The studies have reported that 30–60 % of rheumatoid arthritis patients have anemia which is both iron deficiency and anemia of chronic disease (ACD) type [179,180]. Iron accumulation occurs in rheumatoid arthritis patient's synovial membrane and synovial fluid [181] while serum iron level is decreased in RA patients compared to a control group that is attributable to hepcidin production [182]. Another study has revealed that RA patients with high disease activity have higher levels of iron in the synovial fluid than in patients with moderate disease activity [181]. IO in synovial fluid and synovial cells (e.g., fibroblast-like synoviocytes (FLSs), monocytes) can contribute to ROS generation by the Fenton reaction. Several studies have shown that ROS can play a part in RA development by multiple signal pathway activation (e.g., MAPK, PI3K-Akt, and NF-κB) [183,184], and induction of apoptosis in chondrocytes which results in cartilage damage by inhibition of the interaction between growth factors and chondrocytes [185]. Besides, ROSs and extra lipid oxidation can result in abnormal proliferation of FLSs in RA [123]. In addition, IO can directly lead to bone destruction and osteoporosis by induction of osteoblast apoptosis and promotion of osteoclast differentiation [186]. Furthermore, it has been revealed that NETs trigger a RANKL-independent form of osteoclastogenesis of monocytes [187], therefore, due to decreased levels of serum iron in RA patients, which can induce NETosis, ID may indirectly contribute to the osteoclastogenesis and RA pathogenesis.

IO and overproduction of ROSs and reactive nitrogen species (RNSs) have been involved in somatic gene mutations such as susceptible p53 tumor suppressor gene mutation. Mutant p53 has been detected in synovial tissue and cultured synoviocytes of RA patient's joints [188]. In the case of RA, the dominant p53 mutation is a transition missense mutation [189,190] and results in dysfunctional p53 protein production [188]. Dysfunction of p53 seems to be involved in the promotion of inflammation, HIF-1α stabilization and angiogenesis, resistance to apoptosis in RA-FLSs, and defective apoptotic body clearance [188].

According to the mentioned data synovial iron chelation can inhibit ROS production and osteoclastogenesis resulting in RA regression.

## 8 Role of iron in systemic lupus erythematosus (SLE) pathogenesis

ACD is the most common type of anemia in SLE patients [191,192]. A study has revealed increased serum FT levels in SLE patients compared with healthy people [192]. Although the level of FT correlates with disease activity in SLE patients, FT is an acute-phase reactant protein that confounds this association [2]. The pro-inflammatory state in SLE contributes to functional iron deficiency due to hepcidin production. Abnormal iron metabolism can impair normal mitochondrial function and bioenergetics in the effector cells such as CD4^+^ T cells and monocytes. CD4^+^ T cells and monocytes cultured in iron-deficient conditions showed abnormal mitochondrial function and elevated ROS production [193]. Increased undesirable mitochondrial ROS production has been seen in the T cells derived from SLE patients and has been identified in higher levels in SLE patients with higher disease activity [194]. This elevated ROS may induce signals of apoptosis and ultimately result in increased cellular debris which can be a target for autoantibody production [195]. The change in bioenergetics of monocytes can fail apoptotic debris clearance which is a hallmark of SLE [3]. Also, ROSs can directly damage DNA and this may be a mechanism through which autoantibodies form against DNA antigens [196]. In addition, it has been reported that intracellular iron is increased in lupus patient CD4^+^ T cells and intracellular IO promotes pathogenic Tfh differentiation, GC B cell expansion, and autoantibody production in lupus-prone mice [197]. A study has shown that iron accumulation occurs in SLE patients' kidneys resulting in lupus nephritis (LN, the most common end-organ manifestation of SLE) and iron chelation can postpone the onset of proteinuria and reduce BUN [198]. The proximal tubular epithelial cells (PTECs) in a SLE/LN mouse model showed decreased expression of TfR1 and increased expression of FT suggesting iron accumulation in PTECs [198]. This iron accumulation can lead to ROS generation and lipid peroxidation via the Fenton reaction and aggravate the inflammatory phenotype of PTECs ultimately resulting in tubulointerstitial injury and renal failure [4]. Another study has mentioned that mitochondrial ROS, through induction of NETosis, can contribute to the formation of the lupus phenotype [199]. Therefore, elevated ROS due to abnormal iron metabolism may have a role in the development of the lupus phenotype. Besides, iron accumulation and ferroptosis seem to be important factors contributing to the induction of pathogenic cell phenotypes involved in SLE pathogenesis such as T cells, B cells, macrophages, and neutrophils. Iron accumulation can lead to DAMP release and inflammation in various cell types by induction of ferroptosis and ROS production consequently resulting in autoantibody production and pro-inflammatory cytokine secretion in SLE [200]. So, iron chelation may prevent ROSs, autoantibody production, and LN ultimately preventing lupus progression.

Recent studies highlight the therapeutic potential of targeting iron metabolism in SLE [201]. Deferiprone, an iron chelator, have been shown to delay albuminuria and reduce blood urea nitrogen in lupus models, while hepcidin treatment ameliorates nephritis severity [202]. Liproxstatins, through ferroptosis inhibition, help regulate B-cell differentiation and plasma cell formation, improving renal injury and lupus symptoms [203,204]. Similarly, hepcidin exhibited therapeutic promise by reducing the course and severity of nephritis in aged mice with systemic autoimmunity and proteinuria [205]. Several iron metabolism-targeted drugs, including NAC and DHA, are under clinical investigation for SLE (NCT03396393, NCT00775476). These agents aim to reduce oxidative stress and immune dysregulation while preserving iron homeostasis. However, balancing iron modulation with anemia risks remains a challenge, emphasizing the need for precise therapeutic strategies.

## 9 Role of iron in systemic sclerosis (SSc) pathogenesis

The evidence has reported that there is increased hepcidin in the serum of SSc patients compared with healthy controls. Also, among SSc patients, there are increased prevalence of ID in patients with pulmonary hypertension (SSc-PH) compared with patients without pulmonary hypertension (SSc-non-PH) [206]. It is worth noting that gastrointestinal (GI) manifestations such as GAVE (gastric antral vascular ectasia; a rare GI manifestation of SSc characterized by hemorrhage and unique endoscopic appearance) also called "watermelon stomach" can play a part in iron loss, defective iron uptake, and ultimately ID in SSc patients [207]. On the other hand, it has been revealed that erythrocytes are deposited in tissues in SSc (results from micro vasculopathy in SSc); and subsequently, the resident endothelial cells, fibroblasts, and infiltrating leukocytes uptake their iron which may lead to the production of ROSs in these cells through the Fenton reaction [208]. The experiments have shown that iron may increase TGF-β production by mesenchymal cells [209]. In addition, the reports suggest that high intracellular iron, through ROS production, can induce endothelial-mesenchymal transition (EndMT) [210,211], fibroblast to myofibroblast transition [64,212], which are involved in fibrosis progression and can promote immune cell activation [66,[213], [214], [215]]. Therefore, iron uptake by endothelial cells, fibroblasts, and infiltrating leukocytes can involve intracellular IO and contribute to SSc pathogenesis through the enhancement of fibrosis. Therefore, iron chelation by prevention of fibrosis can contribute to SSc regression.

Moreover, Pulmonary arterial hypertension (PAH) is a leading cause of mortality in patients with systemic sclerosis (SSc). A study highlighted a patient with SSc-associated PAH who exhibited progressive dyspnea and elevated pulmonary artery systolic pressures (PASPs). Following treatment with ferritin-targeted iron infusions, the patient's symptoms resolved, and PASPs normalized [216].

## 10 Role of iron in ankylosing spondylitis (AS) pathogenesis

Ankylosing Spondylitis (AS) is an inflammatory joint disease of the spine without the presence of RF characterized by osteoproliferation, bone growth, and fusion [217]. The most common form of anemia in AS patients is ACD [218]. The reports have shown that serum hepcidin levels are higher in AS patients compared with healthy people and serum hepcidin level is associated with disease activity in AS patients [219]. Another study reported increased intracellular iron in polymorphonuclear cells (PMNs) and platelets of AS patients [220]. Although there is no direct evidence implicating lipid peroxidation in AS patients, several studies have shown enhancement of oxidative stress and ferroptosis markers in patients with AS and AS mice models [[221], [222], [223], [224], [225]]. Despite RA, in AS pathologic osteogenesis occurs and iron supplementation may result in the induction of osteoclastogenesis and AS regression.

The therapeutic landscape of AS includes nonsteroidal anti-inflammatory drugs (NSAIDs), conventional synthetic disease-modifying antirheumatic drugs (csDMARDs), and steroid-based treatments. Additionally, various biological agents such as tumor necrosis factor inhibitors (TNFi), interleukin-17 (IL-17) inhibitors, and Janus kinase (JAK) inhibitors have been approved for AS treatment. Emerging therapies, including anti-IL-6 receptor (anti-IL-6Ra) antibodies and anti-granulocyte-macrophage colony-stimulating factor (anti-GM-CSF) antibodies, have also shown potential in clinical investigations [226]. Despite advancements in AS management, there is a notable gap in research on iron-based therapies for this condition. This approach may complement existing therapeutic strategies, addressing inflammation and immune dysregulation while mitigating disease progression. Further studies are needed to explore the potential of iron-related interventions in AS management.

## 11 Role of iron in gout pathogenesis

Some studies mention the role of iron in gout. A study has shown that urate crystals can form a complex with iron *in vitro* and give rise to stimulation of oxidative stress, granulocytes, and complement activation and some of these conditions are improvable following iron chelation therapy [227]. A study has revealed that serum iron and FT levels were positively correlated with the number of gout attacks, and serum FT was higher in gout patients than in healthy controls [228]. On the other hand, a study suggests that the mechanism by which iron contributes to gout may be through xanthine oxidase (XO), which is a uric acid-producing enzyme and is involved in ROS production too. The underlying reason is that iron can induce the expression and activation of XO and in line with this, iron chelator DFO reduces XO activity [229].

## 12 Conclusion

Iron is a vital factor for the physiological function of the cells in lots of organisms whether immune cells or non-immune cells. Hence, iron homeostasis always is regulated by physiological mechanisms but this homeostasis can be impaired because of inflammatory conditions such as rheumatic diseases. Although this abnormal iron homeostasis can be of various types, the most common form is functional iron deficiency or ACD caused by inflammation in rheumatic diseases. ACD usually leads to the circulating iron unavailability and retention of iron in the stores (the iron-storing cells and tissues). This accumulated iron in the cells and tissues can contribute to the progress and development of rheumatic diseases containing RA, SLE, SSc, AS, and gout through the several mechanisms involved in the pathogenesis of these diseases such as the generation of the ROSs and contributing to the fibrosis, inflammation, abnormal bone homeostasis, NETosis and cell senescence.

In the future, further investigations in the field of iron metabolism in rheumatic diseases and a precise understanding of the impact of this metal on the pathogenesis of these conditions can lead to the introduction of new therapeutic strategies to improve these rheumatic disorders by targeting abnormal iron metabolism.

## CRediT authorship contribution statement

**Aliakbar Givian:** Writing – original draft. **Amin Azizan:** Writing – original draft. **Ahmadreza Jamshidi:** Supervision. **Mahdi Mahmoudi:** Supervision. **Elham Farhadi:** Conceptualization.

## Ethics approval

Not applicable.

## Consent to participate

Not applicable.

## Consent for publication

Not applicable.

## Availability of data and materials

Not applicable.

## Funding

This research did not receive any specific grant from funding agencies in the public, commercial, or not-for-profit sectors.

## Declaration of competing interest

The authors declare that they have no known competing financial interests or personal relationships that could have appeared to influence the work reported in this paper.

## Data Availability

No data was used for the research described in the article.
